# Dynamic trajectories of inflammatory biomarkers and post-stroke cognitive impairment: a comprehensive review of neuroimmune mechanisms, longitudinal modeling, and clinical translation

**DOI:** 10.3389/fneur.2026.1903189

**Published:** 2026-08-20

**Authors:** Hongyu Hao, Hongmin Ding, Jiayu Zhang, Peiyuan Lv

**Affiliations:** 1 Department of Neurology, Hebei General Hospital, Shijiazhuang, Hebei, China; 2 Hebei Provincial Key Laboratory of Cerebral Networks and Cognitive Disorders, Shijiazhuang, China

**Keywords:** acute ischemic stroke, inflammatory biomarkers, longitudinal trajectories, Neuroinflammation, post-stroke cognitive impairment, vascular cognitive impairment

## Abstract

Post-stroke cognitive impairment (PSCI) affects 30–50% of acute ischemic stroke survivors within the first year, yet the prognostic relevance of dynamic inflammatory trajectories remains incompletely defined. This narrative review synthesizes clinical and mechanistic evidence linking temporally evolving inflammatory biomarkers to vascular cognitive impairment after acute ischemic stroke. A structured evidence search of PubMed, MEDLINE, Embase, and Google Scholar from database inception to June 2026 prioritized longitudinal inflammatory-biomarker cohorts, trajectory-modeling analyses, machine-learning prediction studies, and mechanistic literature on neuroimmune injury. The available evidence indicates that post-stroke inflammation follows a multiphasic course, including hyperacute activation, acute peaking, subacute resolution, and, in a subset of patients, persistent low-grade inflammation. Delayed resolution or persistent elevation of interleukin-6, C-reactive protein, and the neutrophil-to-lymphocyte ratio appears to predict PSCI more accurately than single admission measurements. Mechanistically, sustained inflammatory signaling may contribute to chronic microglial activation, mCRP/VE-cadherin-mediated blood–brain barrier disruption, hippocampal and Papez-circuit vulnerability, and secondary neurodegeneration. Trajectory-based modeling, including latent class approaches and machine learning, may therefore improve PSCI risk stratification and support personalized follow-up and anti-inflammatory prevention trials. Larger prospective cohorts with standardized sampling and cognitive assessment are required to validate these translational applications.

## Introduction

Acute ischemic stroke (AIS) stands as a catastrophic cerebrovascular event characterized by high global incidence, debilitating disability rates, and high mortality, imposing an immense socio-economic and public health burden worldwide. In recent decades, therapeutic paradigms for acute-phase AIS have advanced dramatically, driven by the widespread implementation of intravenous thrombolysis and mechanical endovascular thrombectomy. While these hyperacute reperfusion strategies have significantly increased survival rates and mitigated gross motor deficits, a substantial proportion of stroke survivors face insidious, long-term cognitive sequelae. Post-stroke cognitive impairment (PSCI), which affects up to 30–50% of stroke survivors within the first year (1), impairs executive functioning, memory, processing speed, and attention, ultimately hindering successful neurological rehabilitation and social reintegration. Notably, a 2024 prospective study found that even with modern state-of-the-art stroke care, up to 69.3% of patients presented with PSCI in the acute phase (2).

The pathophysiological orchestrators of PSCI are highly complex, involving a multi-faceted interplay between localized ischemic tissue necrosis, structural and functional disruption of central cerebral networks (such as the Papez circuit) (3), chronic cerebral hypoperfusion (4, 5), and oxidative stress. At the intersection of these diverse injury pathways lies neuroinflammation, a ubiquitous process that acts as a double-edged sword throughout the lifespan of a stroke (6). In the hyperacute phase, localized brain ischemia triggers an immediate, profound immune response characterized by microglial activation and the release of cytotoxic pro-inflammatory cytokines (7, 8), and the recruitment of peripheral leukocytes. Although this initial inflammatory cascade is crucial for clearing cellular debris and initiating tissue remodeling, its dysregulation can trigger a secondary “inflammatory storm.” This storm, which is strongly associated with PSCI risk (9), leads to widespread synaptic pruning, blood–brain barrier (BBB) breakdown, and secondary neurodegeneration in key cognitive hubs, particularly the hippocampus (10, 11), even in areas distant from the primary focal infarct.

Historically, research on stroke-related inflammatory biomarkers has focused on cross-sectional, static evaluations—specifically, measuring systemic markers such as C-reactive protein (CRP), high-sensitivity CRP (hs-CRP), interleukin-6 (IL-6), and the neutrophil-to-lymphocyte ratio (NLR) at a single time point, usually at emergency admission or early during hospitalization (9, 12). While these baseline values provide raw indices of stroke severity, they are highly susceptible to acute physiological stress, comorbid systemic infections, or therapeutic interventions (13). Consequently, they fail to reflect the dynamic temporal evolution of the immune response, which naturally progresses from acute activation to subacute resolution and chronic, low-grade persistence (14). In contrast, longitudinal studies have shown that the temporal trajectory of inflammatory markers—including the rate of resolution, time-to-peak, and the presence of persistent subacute elevation—varies widely among individuals. This heterogeneity is highly predictive of long-term neurocognitive recovery or decline, such as post-stroke cognitive impairment (PSCI) (9, 15, 16).

With recent advances in longitudinal data analysis and computational biology, statistical techniques such as latent class growth analysis (LCGA) and growth mixture modeling (GMM) have been applied to characterize these heterogeneous dynamic inflammatory trajectories (17, 18). Furthermore, machine learning models (such as Extreme Gradient Boosting [XGBoost] and Random Forest) are increasingly used to analyze complex, high-dimensional longitudinal datasets, offering superior predictive power over traditional logistic regression for outcomes like PSCI (16, 19, 20). These analytical tools allow researchers to classify patients into distinct inflammatory trajectory subtypes, providing a more robust framework for predicting PSCI risk.

To establish a solid epidemiological foundation, this review integrates findings from major prospective cohorts, notably the “Norwegian Cognitive Impairment After Stroke” (Nor-COAST) study, which has delineated distinct long-term cognitive trajectories and identified associations between acute-phase plasma inflammatory biomarkers (e.g., interleukin-6, terminal complement complex) and worse cognitive outcomes up to 36 months post-stroke (21, 22). Additionally, we incorporate evidence from longitudinal studies highlighting that the dynamic behavior of plasma inflammatory markers over several months is a stronger predictor of cognitive impairment than single baseline measurements alone (23, 24). We also explore the potential of Mendelian randomization (MR) to establish causal links between genetically determined inflammatory pathways and cognitive decline, helping to distinguish whether persistent inflammation is a direct driver of post-stroke cognitive impairment (PSCI) or a secondary marker of severe brain tissue injury (25, 26).

This narrative review synthesizes the molecular, cellular, and computational landscapes of post-stroke inflammatory trajectories. We begin by examining the pathophysiological mechanisms of ischemia–reperfusion injury, neuro-immune crosstalk, and the “inside-out” and “outside-in” pathways of blood–brain barrier (BBB) disruption—focusing on the disruptive interaction between monomeric CRP (mCRP) and vascular endothelial cadherin (VE-cadherin), which compromises endothelial adherens junctions and barrier integrity (27). We then compare advanced statistical and machine learning methods for modeling biomarker trajectories over time. Finally, we analyze the clinical evidence linking dynamic inflammatory trajectories to long-term cognitive outcomes and discuss how these insights can be translated into personalized, trajectory-guided clinical interventions to prevent and treat PSCI.

## Search strategy and evidence selection for this narrative review

To ensure methodological rigor, comprehensiveness, and transparency, this narrative review was supported by a structured, transparent evidence-search strategy informed by established review-reporting principles (28). We conducted a high-sensitivity literature search across electronic databases including PubMed, MEDLINE, Embase, and Google Scholar from database inception through June 2026. The search strategy combined Medical Subject Headings (MeSH) terms and free-text keywords: (“stroke” OR “cerebral infarction” OR “acute ischemic stroke”) AND (“inflammation” OR “inflammatory biomarkers” OR “C-reactive protein” OR “interleukin-6” OR “neutrophil-to-lymphocyte ratio” OR “monomeric CRP” OR “VE-cadherin”) AND (“trajectory” OR “longitudinal modeling” OR “growth curve” OR “latent class growth analysis” OR “growth mixture model” OR “machine learning” OR “XGBoost”) AND (“cognitive impairment” OR “dementia” OR “post-stroke cognitive impairment” OR “PSCI” OR “long-term cognitive outcome”). Only peer-reviewed articles written in English were included. Priority was assigned to high-impact prospective cohort studies (e.g., Nor-COAST, STROKOG, and ICONS), randomized controlled trials, and studies employing advanced longitudinal modeling or machine learning algorithms published within the last 5 years (2020–2026), alongside foundational mechanistic and methodological literature.

This search was not designed to generate a PRISMA flow diagram or a meta-analysis; instead, it was used to identify mechanistic, longitudinal-modeling, clinical-prediction, and translational evidence relevant to the review objectives. The principal clinical cohorts and prediction studies included in this review are summarized in Table 1, while the methodological comparison of trajectory-analysis approaches is presented in Table 2. The following sections therefore synthesize mechanistic, modeling, and clinical evidence thematically, rather than reporting a PRISMA-style study-selection result.

**Table 1:** Landmark longitudinal cohort studies modeling dynamic trajectories of inflammatory biomarkers and post-stroke cognitive outcomes.

Study cohort/author (year)	Sample size (*N*)	Inflammatory markers tracked	Sampling time points	Trajectory modeling methodology	Key neurocognitive & clinical findings	Ref
Nor-COAST cohort Sandvig et al. (21)	466	Plasma TCC, TNF, IL-1β, IL-1ra, IL-6, IL-8, MIP-1*α*, neopterin, QA, Pic, PAr, and HKr	Acute phase (median Day 4 [IQR 3–6]) and 3 months post-stroke	Group-based trajectory modeling (GBTM) of MoCA; Multinomial logistic regression (RRR) and mixed linear regression	Identified 3 MoCA trajectories over 36 months: “Low & declining” (11%), “Moderate & stable” (33%), and “High & increasing” (56%). High acute TCC, IL-6, MIP-1α, neopterin, QA, and PAr significantly predicted the “Low & declining” trajectory. Acute TNF and IL-8 predicted progressive 36-month MoCA decline, especially in those with pre-stroke cognitive impairment.	(21)
Nor-COAST cohort Sandvig et al. (22)	455	Plasma TCC and 20 cytokines (including IL-1β, IL-1Ra, IL-6, IL-7, IL-8, MIP-1α, TNF)	Baseline (acute phase), 3 months, and 18 months post-stroke	Linear mixed-effects modeling of MoCA scores adjusted for age and sex	Demonstrated that elevated acute-phase concentrations of 7 biomarkers (TCC, IL-6, MIP-1α, IL-1Ra, TNF, IL-7, IL-8) are associated with lower MoCA scores at 36 months. Baseline TCC, IL-6, and MIP-1α showed persistent negative associations across all follow-ups (3, 18, and 36 months), indicating that early systemic inflammation has a long-term impact on cognitive recovery.	(22)
ICONS study Wang et al. (54)	1,003	Fasting serum Interleukin-6 (IL-6)	Acute phase (within 24 h of admission)	Multivariable logistic regression (stratified by IL-6 quartiles)	Of 1,003 Chinese ischemic stroke or TIA patients, 23.73% developed cognitive decline (≥2-point MoCA reduction from month 3 to year 1). Elevated baseline IL-6 was an independent predictor of 1-year cognitive decline, with patients in the highest quartile (≥4.29 ng/L) exhibiting a 1.95-fold increased risk (adjusted OR = 1.95, 95% CI: 1.13–3.38, *p* = 0.0167).	(54)
STROKOG collaboration Lo et al. (66)	1,149	Standardized global and domain-specific cognitive z-scores	Baseline (median 3.6 months), 1 year (T2), and long-term (median 3.2 years [T3])	Latent Class Growth Analysis (LCGA) and linear mixed-effects models	Identified 3 stable 1-year cognitive trajectories: Low-performance (17%), Medium-performance (48%), and High-performance (35%). Class membership strongly predicted T3 global cognition. Risk factors for membership in the Low-performance group included advanced age, lower education, diabetes (RRR = 3.78), large-artery stroke (RRR = 2.77), and moderate/severe stroke (RRR = 3.17).	(66)
RIR cohort Gang et al. (82)	172	hs-CRP, LDL-C, WBC, Neutrophils, Lymphocytes, Monocytes	Fasting blood within 24 h of admission (median 31.6 h)	Multivariable logistic regression stratified by Residual Inflammatory Risk (RIR)	At 6 months, 33.7% of patients developed PSCI (MoCA < 22). Patients with RIR (defined as LDL-C < 2.6 mmol/L and hs-CRP ≥ 2.0 mg/L) had a significantly higher risk of PSCI (OR = 4.496, 95% CI: 1.157–17.477, *p* = 0.030). Combined RCIR further increased the risk of cognitive deterioration (OR = 7.357, *p* = 0.002).	(82)
XGBoost prediction study Yang et al. (110)	261	Serum VE-Cad, FGF21, CRP, HCY, UA, HbA1c, and BUN/SCr ratio	Baseline assessment in AIS patients enrolled within 7 days of symptom onset	LassoCV for feature selection; Extreme Gradient Boosting (XGBoost) vs. Logistic Regression (LR) with SHAP explanations	Vascular Endothelial Cadherin (VE-Cad) was identified as the strongest predictor of 3-month PSCI (MoCA < 26). The XGBoost model demonstrated superior predictive performance (validation set AUC = 0.806, sensitivity = 85.0%, accuracy = 73.4%) compared to LR. Individual risk surged when baseline VE-Cad exceeded 13.64 ng/mL and CRP exceeded 10.27 mg/L.	(110)

AIS, acute ischemic stroke; AUC, area under the curve; AUROC, area under the receiver operating characteristic curve; bFGF, basic fibroblast growth factor; GCSF, granulocyte colony-stimulating factor; GMM, growth mixture modeling; HKr, HK ratio (3-hydroxykynurenine/(kynurenic acid + anthranilic acid + xanthurenic acid + 3-hydroxyanthranilic acid) * 100); hs-CRP, high-sensitivity C-reactive protein; IL-6, interleukin-6; LCGA, latent class growth analysis; LGCM, latent growth curve modeling; MMP-9, matrix metalloproteinase-9; MoCA, Montreal Cognitive Assessment; NLR, neutrophil-to-lymphocyte ratio; OR, odds ratio; PAr, PA ratio (4-pyridoxic acid / (pyridoxal + pyridoxal 5′-phosphate)); Pic, picolinic acid; PSCI, post-stroke cognitive impairment; QA, quinolinic acid; RCIR, residual cholesterol and inflammatory risk; RIR, residual inflammatory risk; RRR, relative risk ratio; TCC, terminal C5b-9 complement complex; TIA, transient ischemic attack; TNF-α, tumor necrosis factor-alpha.

**Table 2:** Multidimensional comparison of traditional statistical modeling vs. advanced machine learning in Post-Stroke inflammatory trajectory analytics.

Analytical dimension	Traditional latent class statistical models (e.g., LCGA, GMM, GBTM)	Advanced machine learning models (e.g., XGBoost, Random Forest)
Core mathematical philosophy	Parametric-driven; assumes that population-level heterogeneity can be captured by a finite mixture of distinct latent subpopulations (classes) sharing similar growth parameters (intercept, slope).	Non-parametric and data-driven; optimization based on decision trees and gradient boosting, focusing on minimizing loss functions through iterative tree ensembles.
Handling of non-linear trajectories	Requires explicit polynomial specifications (e.g., linear, quadratic, or cubic terms). Struggling with sudden chaotic environmental shifts or secondary infection spikes.	Naturally accommodates highly complex, non-linear multi-dimensional interactions and temporal fluctuations without requiring prior mathematical formulation.
Input variable dimensionality	Limited; primarily restricted to a single or a few parallel biomarker processes to avoid model non-convergence and overfitting.	High; capable of processing hundreds of parallel, high-dimensional inputs including clinical, metabolic, proteomic, and multimodal neuroimaging parameters.
Clinical interpretability	High; provides clear, clinically intuitive visual trajectories (e.g., “rapid-resolution” vs. “persistent-high”) and explicit parameter estimations.	Moderate to low; often regarded as a “black box” though explainable AI tools (such as SHAP values) are increasingly used to reveal feature importance.
Predictive accuracy (AUROC)	Moderate (typically AUROC: 0.70–0.78) for long-term PSCI prediction due to structural constraints of parametric assumptions.	Excellent (typically AUROC: 0.82–0.91) for individualized clinical risk prediction by utilizing multi-temporal inflammatory kinetics.

AI, artificial intelligence; AUROC, area under the receiver operating characteristic curve; GMM, growth mixture modeling; LCGA, latent class growth analysis; ML, machine learning; PSCI, post-stroke cognitive impairment; SHAP, SHapley Additive exPlanations; XGBoost, Extreme Gradient Boosting.

## Pathophysiological mechanisms of inflammatory responses following acute ischemic stroke

### Ischemia–reperfusion injury and the inflammatory cascade

Following the hyperacute occlusion of a cerebral artery, the sudden cessation of localized blood supply deprives the brain parenchyma of vital oxygen and glucose, initiating a cascade of metabolic collapse. Rapid tissue necrosis ensues in the core of the infarct, while cells within the surrounding ischemic penumbra remain biochemically active but highly vulnerable. Although the immediate therapeutic objective in clinical stroke medicine is hyperacute vascular recanalization via intravenous thrombolysis or endovascular mechanical thrombectomy, the physical restoration of cerebral blood flow introduces a secondary, highly destructive phenomenon known as ischemia–reperfusion injury (IRI) (29). The molecular pathology of IRI is characterized by a complex, interconnected network of oxidative stress, mitochondrial dysfunction, intracellular calcium overload, and excitatory amino acid toxicity, with the inflammatory cascade serving as the central orchestrating link that converts localized vascular occlusion into widespread tissue injury (30).

The post-stroke inflammatory cascade begins when necrotic and severely stressed cells in the ischemic core release danger-associated molecular patterns (DAMPs) into the extracellular space (31). High-mobility group box 1 (HMGB1), a prototypical DAMP, is rapidly mobilized and secreted during the hyperacute phase, binding to receptors such as Toll-like receptors (TLR2 and TLR4) and the Receptor for Advanced Glycation Endproducts (RAGE) on resident microglia and endothelial cells, thereby initiating the immune response (32). Upon the reintroduction of oxygenated blood during reperfusion, a massive burst of reactive oxygen species (ROS) is generated by NADPH oxidase (NOX) and mitochondrial respiratory chains (30, 33). This oxidative stress environment activates the intracellular NOD-like receptor protein 3 (NLRP3) inflammasome within microglia and infiltrating macrophages (34). The assembly of the NLRP3 inflammasome leads to the auto-proteolytic cleavage of caspase-1, which subsequently processes the precursor proteins of the highly pyrogenic and pro-inflammatory cytokines interleukin-1β (IL-1β) and interleukin-18 (IL-18) into their mature, biologically active forms (34). These cytokines damage surrounding neurons and prompt neighboring astrocytes to adopt a pro-inflammatory reactive phenotype (35).

**Figure 1:**
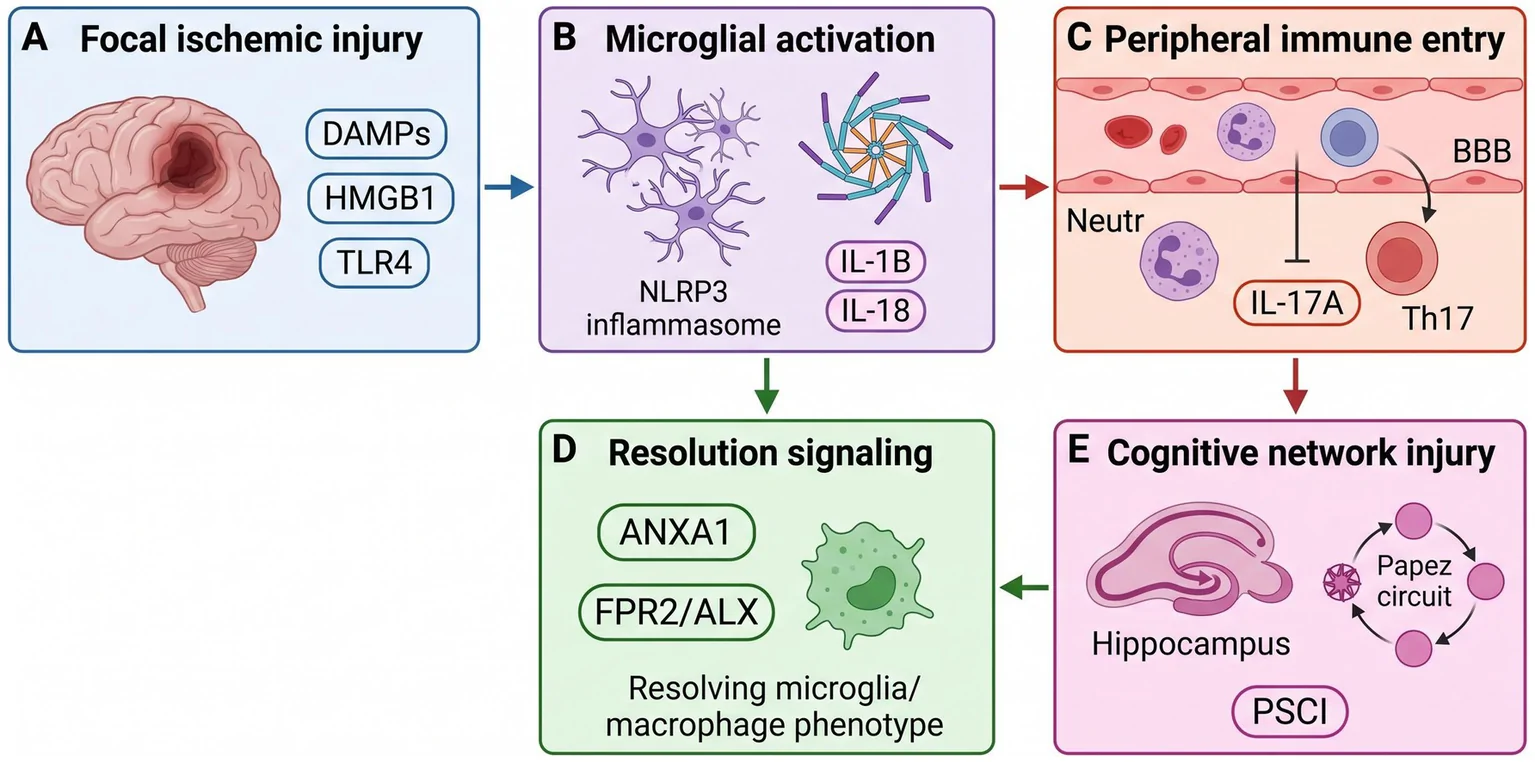
Post-stroke central and systemic inflammatory cascade. **(A)** depicts focal ischemic injury with DAMP/HMGB1/TLR4 signaling; **(B)** activated microglia and NLRP3-associated IL-1β/IL-18 production; **(C)** peripheral immune entry across the BBB, including neutrophil/Th17-related inflammatory responses; **(D)** ANXA1/FPR2-ALX-mediated resolution signaling; and **(E)** hippocampal/Papez-circuit injury relevant to PSCI. Source, software, and provenance: This author-directed conceptual schematic was based on the published literature cited in the corresponding main-text section and mapped in Supplementary file S1. No patient-level data, original experimental images, or quantitative datasets are depicted. An initial visual draft was generated from an author-written text specification using OpenAI gpt-image, version 2.0 (OpenAI Media Service API). The authors independently verified and approved the final scientific content, labels, arrows, and graphical relationships. The complete prompt and materials inventory are provided in Supplementary file S1 and the accompanying plain-text prompt record.

Concurrently, endogenous anti-inflammatory mechanisms attempt to counteract this cascade. Annexin A1 (ANXA1), a calcium-dependent phospholipid-binding protein, acts as a potent anti-inflammatory mediator by binding to the formyl peptide receptor 2 (FPR2/ALX) (36). ANXA1 downregulates the expression of pro-inflammatory cytokines, limits the transmigration of circulating neutrophils into the cerebral parenchyma, and promotes the resolution of inflammation by modulating microglia/macrophage polarization toward an anti-inflammatory phenotype (36). A visual synthesis of focal ischemic injury and DAMP release (Figure 1A), microglial inflammasome activation (Figure 1B), peripheral immune entry (Figure 1C), resolution signaling (Figure 1D), and cognitive-network injury relevant to PSCI (Figure 1E) is provided in Figure 1.

At the intracellular level, several signaling networks dictate the balance between survival and programmed cell death. The phosphatidylinositol 3-kinase/protein kinase B (PI3K/Akt) signaling pathway serves as a vital neuroprotective mechanism; its activation promotes cell survival by inactivating pro-apoptotic targets and suppressing the transcriptional activity of pro-inflammatory factors (37). Simultaneously, a delicate regulatory crosstalk exists between the nuclear factor erythroid 2-related factor 2 (Nrf2) and the nuclear factor kappa B (NF-κB) signaling pathways (38). Under physiological conditions, Nrf2 orchestrates the antioxidant response by binding to antioxidant response elements (ARE) and promoting the transcription of detoxifying enzymes (39). Under the stress of IRI, Nrf2 activation directly suppresses the nuclear translocation of NF-κB, thereby inhibiting the downstream transcription of pro-inflammatory cytokines, chemokines, and cell adhesion molecules (38, 40). When Nrf2 activity is overwhelmed or genetically deficient, unchecked NF-κB activation sustains a self-amplifying cycle of neuroinflammation and cell death (36).

### The role of neuro-immune interactions in stroke progression

Ischemic stroke is not merely a localized neurological event; it triggers a profound bidirectional communication between the central nervous system (CNS) and the peripheral immune system, fundamentally shaping clinical progression and recovery. Following blood–brain barrier (BBB) breakdown, this neuro-immune interaction allows peripheral immune cells to rapidly infiltrate the brain parenchyma (41). Within hours of stroke onset, neutrophils migrate across the compromised vascular wall, guided by chemokines such as CXCL1 and CXCL2 (42), and release matrix metalloproteinases (specifically MMP-9), reactive oxygen species, and neutrophil extracellular traps (NETs), which exacerbate microvascular obstruction and secondary tissue damage (43, 44). This is followed by the infiltration of peripheral monocytes, which differentiate into macrophages and, along with resident microglia, populate the ischemic boundary zone (45). Concurrently, adaptive immune cells are recruited; helper T cells, particularly T helper 17 (Th17) cells, infiltrate the brain and release the potent pro-inflammatory cytokine interleukin-17A (IL-17A), which exacerbates chemokine expression and microglial activation, worsening long-term neurological and cognitive deficits (46, 47).

Simultaneously, the damaged brain sends reciprocal signals to the periphery via the activation of the sympathetic nervous system (SNS) and the hypothalamic–pituitary–adrenal (HPA) axis, inducing a state known as stroke-induced immunosuppression (SIIS) (48). Excessive release of catecholamines and glucocorticoids triggers the rapid apoptosis of splenic and peripheral B and T lymphocytes, while shifting the remaining immune cells toward an anti-inflammatory, Th2-dominated phenotype (48, 49). While SIIS is an evolutionary adaptation designed to limit autoimmune responses against brain-specific antigens, it renders patients highly vulnerable to post-stroke infections (such as pneumonia and urinary tract infections), which can trigger systemic inflammatory spikes and worsen long-term cognitive outcomes (48).

As the stroke progresses from the acute to the subacute and chronic recovery phases, the functional phenotypes of resident microglia and infiltrating macrophages undergo dynamic changes. Microglia polarize along a continuous spectrum, historically simplified into the classical pro-inflammatory M1 phenotype and the alternative anti-inflammatory M2 phenotype (50). During the first few days post-stroke, the M1 phenotype dominates, releasing neurotoxic cytokines (IL-1*β*, TNF-*α*, IL-6) and maintaining neuroinflammation. However, during the subacute repair phase, microglia gradually transition toward the M2 phenotype, characterized by the expression of CD206, Arginase-1, and the secretion of transforming growth factor-beta (TGF-β) and brain-derived neurotrophic factor (BDNF) (50, 51). M2 microglia facilitate tissue remodeling, promote axonal sprouting, support synaptic plasticity, and clear myelin debris, illustrating that neuroinflammation is a key driver of both injury and repair (50).

### Biological characteristics and clinical significance of inflammatory markers

Evaluating systemic inflammatory markers provides clinicians with accessible tools for risk stratification and prognostic assessment in acute ischemic stroke (AIS). Among these, C-reactive protein (CRP), high-sensitivity CRP (hs-CRP), interleukin-6 (IL-6), and the neutrophil-to-lymphocyte ratio (NLR) have been widely investigated. CRP is an acute-phase reactant synthesized primarily by hepatocytes under the transcriptional control of IL-6 (52). Clinically, elevated admission hs-CRP serves as an independent predictor of stroke severity, functional disability, and mortality. A 2023 meta-analysis of nine studies involving 3,893 participants confirmed that peripheral CRP concentrations in the acute stroke phase are significantly higher in patients who develop cognitive decline post-stroke compared to those who do not (53). Moreover, persistently elevated hs-CRP in the chronic phase is associated with cognitive decline, suggesting that low-grade systemic inflammation contributes to vascular cognitive impairment (9).

IL-6 is a pleiotropic cytokine produced by immune cells, endothelial cells, and astrocytes in response to ischemic injury. Serum IL-6 levels rise rapidly after stroke, correlating with infarct volume, neurological deficits, and poor functional outcomes. In elderly populations, elevated systemic IL-6 is a robust biological predictor of cognitive decline, highlighting its role in accelerating neurodegenerative pathways. A 2022 study indicated that elevated IL-6 levels were independently associated with cognitive impairment in patients with ischemic stroke and transient ischemic attack (54).

The NLR, which divides the absolute neutrophil count by the lymphocyte count, reflects the balance between active innate inflammation and stress-induced adaptive immunosuppression. An elevated NLR at admission is independently associated with large vessel occlusion, severe neurological deficits (NIHSS score), and poor short-term outcomes. A 2024 multicenter study of 1,082 patients with anterior circulation large vessel occlusion found that admission NLR correlated significantly with baseline and 24-h NIHSS scores, reflecting stroke severity, though it did not independently predict 90-day outcomes in patients achieving successful revascularization (55). Other studies have identified specific NLR thresholds; for instance, a postoperative NLR ≥ 6.15 predicts malignant cerebral edema following endovascular treatment (56).

Recent advances have highlighted the role of novel, synergistic molecular markers in the pathogenesis of post-stroke cognitive decline, particularly the interaction between monomeric CRP (mCRP) and vascular endothelial cadherin (VE-cadherin). While pentameric CRP (pCRP) is the primary circulating form, its deposition on damaged endothelial membranes triggers a conformational shift into its highly pro-inflammatory monomeric form, mCRP. Research has demonstrated that mCRP binds directly to the extracellular domain of VE-cadherin, an essential cell adhesion molecule that maintains endothelial cell-to-cell junctions. This interaction triggers the phosphorylation and internal degradation of VE-cadherin, disrupting the structural integrity of the blood–brain barrier. The resulting endothelial disassembly facilitates the infiltration of peripheral immune cells, exacerbating localized neuroinflammation and contributing to post-stroke cognitive impairment. A 2024 study in an ApoE−/− murine model showed that mCRP deposits in brain microvessels and co-localizes with endothelial markers, indicating its role in disrupting the neurovascular unit and increasing blood–brain barrier permeability (57). Furthermore, another 2024 study revealed that mCRP, particularly in the presence of ApoE4, disrupts endothelial adherens junctions by affecting the CD31-VE-cadherin interaction and actin cytoskeleton, providing a mechanistic link to cerebrovascular dysfunction and cognitive decline risk (27).

**Figure 2:**
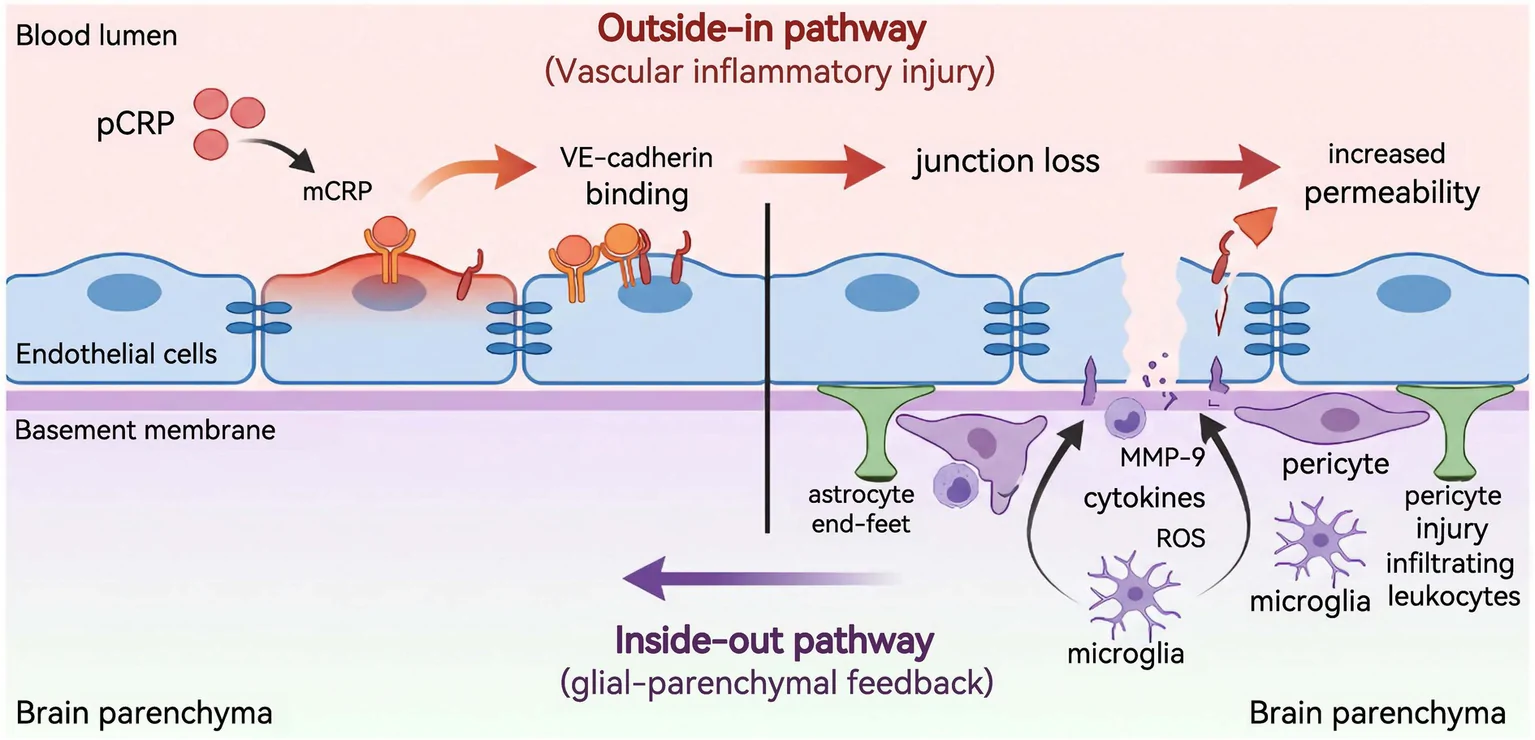
mCRP-VE-cadherin axis and BBB disruption after stroke. The upper luminal compartment summarizes the outside-in pathway: Circulating pCRP deposits on injured endothelial membranes, dissociates to mCRP, binds VE-cadherin, and causes adherens-junction loss and increased BBB permeability. The lower parenchymal compartment summarizes the inside-out pathway: Activated microglia, astrocyte end-feet, pericyte injury, infiltrating leukocytes, MMP-9, cytokines, and ROS feed back on endothelial cells and aggravate BBB disruption. Source, software, and provenance: This author-directed conceptual schematic was based on the published literature cited in the corresponding main-text section and mapped in Supplementary file S1. No patient-level data, original experimental images, or quantitative datasets are depicted. An initial visual draft was generated from an author-written text specification using OpenAI GPT-image, version 2.0 (OpenAI Media Service API). The authors independently verified and approved the final scientific content, labels, arrows, and graphical relationships. The complete prompt and materials inventory are provided in Supplementary file S1 and the accompanying plain-text prompt record.

Figure 2 illustrates neurovascular-unit BBB disruption after stroke. The luminal outside-in pathway begins with pCRP deposition at injured endothelial membranes, dissociation to mCRP, VE-cadherin binding, junction loss, and increased permeability. The abluminal inside-out pathway reflects glial-parenchymal feedback, in which activated microglia, astrocyte end-feet, pericyte dysfunction, infiltrating leukocytes, MMP-9, cytokines, and ROS further destabilize endothelial junctions and amplify BBB leakage.

## Patterns of dynamic changes in inflammatory markers and their measurement methods

### Temporal characteristics of inflammatory markers in the acute phase

Following acute ischemic stroke (AIS), the inflammatory response exhibits distinct temporal dynamics. In the hyperacute phase (0–24 h post-stroke), the initial brain injury triggers a rapid release of pro-inflammatory cytokines. Elevated levels of interleukin-6 (IL-6) and tumor necrosis factor-alpha (TNF-*α*) in the cerebrospinal fluid and peripheral blood have been observed, and these levels show a positive linear correlation with both the time from symptom onset to emergency room presentation and the initial stroke severity (as measured by NIHSS scores) (58). In the subsequent acute phase (days 1–3), the systemic inflammatory response peaks. A key feature is the rapid change in circulating leukocyte profiles, characterized by a significant increase in neutrophils and a relative decrease in lymphocytes, leading to a peak in the neutrophil-to-lymphocyte ratio (NLR) within the first 48–72 h. This elevated NLR at admission is a recognized independent predictor of poorer short-term and 90-day functional outcomes (59, 60). Concurrently, under the transcriptional influence of IL-6, hepatic synthesis of C-reactive protein (CRP) surges. High-sensitivity CRP (hs-CRP) is a prominent acute-phase protein whose levels are closely associated with stroke severity and outcomes (61), typically peaking between 48 and 72 h post-stroke (62).

During the subacute and chronic recovery phases (weeks to months), inflammatory markers generally decline toward baseline in most patients. However, a clinically significant subset of stroke survivors exhibits persistent, low-grade systemic inflammation. This state, sometimes referred to as residual inflammatory risk, is characterized by sustained elevations in markers such as hs-CRP and IL-6 months after the acute event. This persistent inflammation is implicated in the pathogenesis of neuropsychiatric complications, including post-stroke depression and long-term cognitive decline (63).

Furthermore, hyperacute reperfusion therapies, including intravenous thrombolysis and mechanical thrombectomy, can modulate these inflammatory trajectories. Successful recanalization may attenuate the inflammatory cascade, whereas failed reperfusion or reperfusion injury (such as hemorrhagic transformation) can provoke secondary inflammatory spikes. Notably, elevated pre-thrombolysis hs-CRP levels (e.g., >1.6 mg/L) have been associated with a reduced likelihood of a good clinical response to intravenous thrombolysis, highlighting the interplay between inflammation and treatment efficacy (62). These secondary inflammatory events are linked to worse long-term neurological and cognitive outcomes.

### Modeling methods for dynamic inflammatory trajectories

Traditional clinical studies have relied on single-time-point, cross-sectional biomarker assessments at admission, which cannot capture the dynamic evolution of the post-stroke inflammatory response. To analyze longitudinal data and capture individual differences in these biomarkers over time, advanced statistical modeling techniques are required. Growth curve models (GCM) and latent growth models (LGM) treat longitudinal measurements as continuous functions of time, estimating population-level trajectories while accounting for individual variation in both the baseline level (intercept) and the rate of change (slope) (64). In stroke research, these techniques have been applied to model recovery trajectories, such as identifying latent subgroups of post-stroke cognitive function (65, 66).

To capture population heterogeneity, researchers utilize latent class growth analysis (LCGA) and growth mixture modeling (GMM) (67). These techniques assume the population is composed of distinct, unobserved (latent) subgroups, each characterized by a unique trajectory profile. GMM allows for individual variation (random effects) within each class, whereas LCGA (a special case of group-based trajectory modeling, GBTM) assumes all individuals within a class share the same trajectory (68, 69). These modeling techniques have been applied to analyze longitudinal outcomes in stroke cohorts, revealing distinct trajectory classes (e.g., “stable, ““highly variable,” “improving”) that are strongly associated with long-term functional and cognitive outcomes (17, 66). To help researchers navigate the mathematical and clinical paradigms of longitudinal modeling, Table 2 provides a comprehensive, multidimensional comparison of traditional latent class statistical models (e.g., LCGA, GMM, and GBTM) versus advanced non-parametric machine learning frameworks. As compared in Table 2, traditional latent class statistical models are highly parametric-driven, requiring explicit polynomial specifications and being limited in handling high-dimensional parallel processes, which contrasts sharply with the flexible, data-driven, non-parametric optimization of tree-based machine learning architectures.

**Figure 3:**
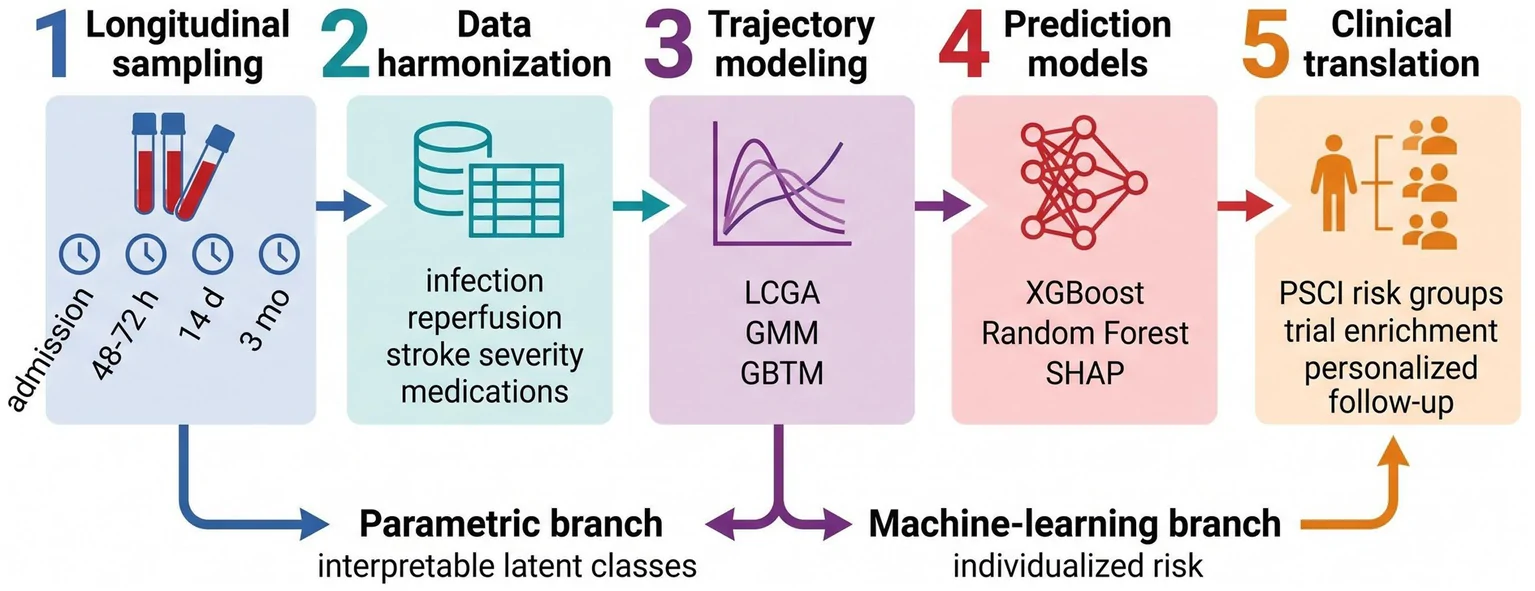
Five-stage workflow for inflammatory-trajectory analysis and PSCI prediction. Stage 1 shows longitudinal biomarker sampling at admission, 48–72 h, 14 d, and 3 months; Stage 2 data harmonization for infection, reperfusion, stroke severity, and medication effects; Stage 3 trajectory modeling using LCGA, GMM, and GBTM; Stage 4 prediction modeling using XGBoost, Random Forest, and SHAP-based interpretation; and Stage 5 clinical translation into PSCI risk grouping, trial enrichment, and personalized follow-up. The lower branch highlights the distinction between parametric latent-class modeling and machine-learning-based individualized risk prediction. Source, software, and provenance: This author-directed conceptual workflow was based on the published methodological and clinical literature cited in the corresponding main-text section and mapped in Supplementary file S1. No patient-level data, original experimental images, cohort-level model output, or quantitative datasets are depicted. An initial visual draft was generated from an author-written text specification using OpenAI gpt-image, version 2.0 (OpenAI Media Service API). The authors independently verified and approved the final scientific content, labels, arrows, and graphical relationships. The complete prompt and materials inventory are provided in Supplementary file S1 and the accompanying plain-text prompt record.

In addition to traditional statistical methods, machine learning (ML) algorithms (such as Extreme Gradient Boosting [XGBoost] and Random Forest) are increasingly used to analyze complex, high-dimensional datasets (70, 71). Unlike GMM and LCGA, which rely on parametric assumptions, ML algorithms are non-parametric and can model complex, non-linear interactions and high-dimensional predictor variables without requiring prior statistical assumptions (Figure 3). As detailed in Table 2, while traditional models offer high clinical interpretability and explicit trajectory groups, advanced machine learning architectures like XGBoost yield significantly superior predictive accuracy for individualized clinical risk prediction by effectively utilizing high-dimensional inputs and capturing complex patterns (72). As shown in Figure 3, Stage 1 covers longitudinal sampling, Stage 2 data harmonization, Stage 3 trajectory modeling, Stage 4 prediction models, and Stage 5 clinical translation.

### Key factors influencing the shape of inflammatory trajectories

The temporal trajectory of post-stroke inflammatory markers is shaped by a complex interaction of clinical, anatomical, and environmental factors. First, patient baseline characteristics are key determinants of the inflammatory profile. Advanced age is associated with “inflammaging,” a chronic, low-grade inflammatory state that predisposes individuals to more severe post-stroke inflammatory responses and slower recovery (73). Pre-existing metabolic comorbidities, particularly type 2 diabetes mellitus, alter the host immune response; hyperglycemia-induced oxidative stress, advanced glycation endproducts, and pre-existing endothelial dysfunction promote a more severe, prolonged inflammatory trajectory following ischemic stroke (74). Baseline stroke severity, measured by the National Institutes of Health Stroke Scale (NIHSS) score at admission, is also strongly associated with the post-stroke inflammatory trajectory; patients with higher initial NIHSS scores typically exhibit higher peak values and slower resolution of inflammatory markers (58).

Second, the anatomical and physiological characteristics of the ischemic lesion influence the inflammatory profile. Infarctions located in critical cognitive networks (such as the hippocampal formation, thalamus, or bi-hemispheric structures) often trigger a more complex inflammatory response, likely due to the disruption of pathways regulating central and autonomic immune homeostasis (75). Large-volume infarcts, typically caused by large vessel occlusions (LVO), release a high concentration of damage-associated molecular patterns (DAMPs), triggering a robust inflammatory response (76).

Third, acute therapeutic interventions can alter the post-stroke inflammatory trajectory. Intravenous thrombolysis (IVT) and mechanical thrombectomy (MT) rapidly restore blood flow, which can limit infarct expansion but may also trigger a transient, reperfusion-induced inflammatory spike. Studies have shown that the post-procedural neutrophil-to-lymphocyte ratio (NLR) trajectory is associated with recanalization status and the risk of symptomatic intracranial hemorrhage, with successful, uncomplicated recanalization leading to a more rapid resolution of systemic inflammatory markers (74, 77).

Finally, the development of post-stroke complications, particularly infections, can modify the inflammatory profile. Post-stroke infections, such as stroke-associated pneumonia (SAP), can trigger a secondary systemic inflammatory spike, interrupting the natural resolution of inflammatory markers (78). Elevated levels of IL-6 and CRP within the first 24 h of admission have been shown to predict the risk of subsequent SAP, illustrating that early alterations in the inflammatory profile can serve as predictors of infectious complications (79).

## Mechanisms linking inflammatory responses to post-stroke cognitive impairment

### Inflammatory storm-induced damage to the hippocampus and cognition-related brain regions

The occurrence of post-stroke cognitive impairment (PSCI) is closely linked to structural and functional damage within key cognitive networks, particularly the hippocampal formation, which is highly vulnerable to both ischemic and inflammatory injury (80). The localized “inflammatory storm” triggered by acute ischemic stroke causes direct and indirect damage to hippocampal neurons and synaptic connections through several key pathways. Elevated concentrations of pro-inflammatory cytokines (including IL-1β, TNF-*α*, and IL-6) exert direct neurotoxic effects, altering neuronal membrane potentials, disrupting intracellular calcium homeostasis, and promoting apoptotic cell death (81, 82). Under physiological conditions, adult neurogenesis persists in the subgranular zone of the dentate gyrus, supporting hippocampal plasticity and memory formation. Following a stroke, the pro-inflammatory microenvironment suppresses neurogenesis by downregulating neurotrophic factors and promoting the differentiation of neural stem cells into glial phenotypes rather than functional neurons (82). In animal models, cognitive decline induced by chronic inflammation is associated with microglial activation, monocyte infiltration, and elevated hippocampal cytokine expression, even in the absence of elevated systemic inflammation, highlighting the role of persistent central neuroinflammation in PSCI (83).

In addition to direct neurotoxicity, the post-stroke inflammatory storm disrupts synaptic plasticity, the neurobiological basis of learning and memory. High levels of TNF-*α* can promote the endocytosis of AMPA receptors and upregulate NMDA receptor activity, leading to excitotoxicity and impairing long-term potentiation (LTP) in hippocampal synapses (81, 82). Furthermore, persistent microglial activation triggers aberrant synaptic pruning. Under the influence of inflammatory signals, microglia upregulate components of the classical complement cascade (specifically C1q and C3), which tag vulnerable synapses. Microglia then engulf and clear these tagged synapses via complement-mediated phagocytic pathways, leading to a loss of synaptic density in the hippocampus and associated cognitive networks (84, 85). These structural changes are accompanied by secondary neurodegeneration within extended limbic networks, including the Papez circuit. Focal cortical or subcortical infarctions can cause remote retrograde or anterograde degeneration of interconnected limbic structures, such as the hippocampus and anterior thalamus, a process potentially driven by axonal transport failure, chronic glial activation, and persistent neuroinflammation (86). Focal hippocampal infarctions, though less common, can cause direct ischemic tissue necrosis, resulting in acute, severe memory impairment (87).

### The persistent role of chronic inflammation in cognitive decline

While the acute post-stroke inflammatory response is a direct reaction to focal tissue injury, a persistent, low-grade systemic and central inflammatory state—chronic neuroinflammation—can continue to drive long-term cognitive decline. Epidemiological studies have demonstrated a clear association between persistent systemic inflammation and long-term cognitive deterioration. In older adult populations, elevated circulating interleukin-6 (IL-6) is consistently associated with accelerated cognitive decline and serves as an independent predictor of dementia risk (88). A meta-analysis of longitudinal studies further supports that elevated systemic inflammatory markers, particularly IL-6, in midlife can predict cognitive decline years later, illustrating that chronic inflammatory exposure has a cumulative, long-term impact on neurocognitive function (88). In stroke survivors, the acute event may accelerate this process, transitioning a subclinical inflammatory state into a chronic, self-sustaining inflammatory loop.

Several mechanisms link chronic systemic inflammation to progressive cognitive decline. First, persistent systemic inflammation is hypothesized to promote endothelial dysfunction and accelerate atherosclerosis in the cerebral microvasculature, though direct human evidence from the past five years specifically linking circulating cytokines to these vascular changes in cognitive decline is limited. Chronic inflammation can upregulate endothelial adhesion molecules (e.g., ICAM-1, VCAM-1), reduce nitric oxide bioavailability, and promote microvascular remodeling, which are believed to contribute to chronic cerebral hypoperfusion, white matter hyperintensities, and microvascular lesions. Second, chronic inflammation can induce systemic and central insulin resistance, thereby impairing neuronal glucose metabolism and the downstream signaling pathways critical for synaptic plasticity and cell survival; however, recent (2020+) primary research directly connecting this mechanism to post-stroke cognitive decline is sparse. Third, and increasingly recognized, chronic inflammation can modulate the gut-microbiota-brain axis (89). Ischemic stroke can induce intestinal dysbiosis, characterized by a loss of microbial diversity and a compromised intestinal barrier. This “leaky gut” allows pathogen-associated molecular patterns (such as lipopolysaccharides, LPS) to enter the systemic circulation, sustaining a systemic inflammatory loop (90). This chronic systemic inflammation can communicate with the central nervous system via humoral and cellular pathways, activating resident microglia and sustaining neuroinflammation, which can accelerate neurodegenerative pathology. Recent studies specifically highlight that post-stroke reduction of beneficial gut bacteria (e.g., butyrate-producers) and increased intestinal permeability contribute to systemic LPS translocation and neuroinflammation, exacerbating cognitive impairment.

### The vicious cycle of blood–brain barrier disruption and neuroinflammation

The blood–brain barrier (BBB) is a specialized structural and functional interface that regulates molecular transport between the systemic circulation and the brain parenchyma, maintaining central nervous system homeostasis. Following an ischemic stroke, the BBB undergoes structural and functional breakdown, which plays a key role in the pathogenesis of post-stroke cognitive impairment (PSCI) (91, 92). In the acute phase, ischemia, hypoxia, and reperfusion trigger the upregulation and activation of matrix metalloproteinases, particularly MMP-9, by endothelial cells, neutrophils, and astrocytes (93, 94). MMP-9 degrades key structural proteins of the extracellular matrix and tight junctions (such as claudin-5, occludin, and ZO-1), increasing paracellular permeability and predisposing the tissue to vasogenic edema and hemorrhagic transformation (94–97). Clinically, elevated serum MMP-9 levels are associated with increased BBB permeability and poor functional outcomes (97, 98).

At the abluminal side of the neurovascular unit, astrocytic end-feet and pericytes provide structural and trophic support for endothelial tight junctions and the basement membrane. After stroke, activated microglia, reactive astrocytes, and infiltrating leukocytes release MMP-9, cytokines, and ROS, weakening pericyte-endothelial and astrocyte-endothelial signaling and amplifying central neuroinflammation. This glial-mural-cell feedback explains why Figure 2 links astrocytic end-feet, pericytes, and central inflammation to progressive BBB disruption.

The structural breakdown of the BBB initiates a self-sustaining pathological loop between neuroinflammation and barrier dysfunction (99). Increased barrier permeability allows circulating pro-inflammatory cytokines (IL-6, TNF-*α*), immunoglobulins, and neurotoxic plasma proteins (such as fibrinogen and albumin) to enter the brain parenchyma (93, 100). The extravasation of fibrinogen is particularly harmful, as its altered conformation can bind to Mac-1 (CD11b/CD18) receptors on microglia and other immune cells, triggering a pro-inflammatory phenotype, reactive oxygen species (ROS) generation, and neurotoxic cytokine release (101). This neuroinflammatory response, in turn, releases additional matrix metalloproteinases, chemokines, and reactive oxygen species, further disrupting tight junction proteins and increasing BBB permeability (99). This “BBB disruption—inflammatory infiltration—neuroinflammation—further BBB disruption” cycle can persist into the chronic phase of stroke recovery, exposing the brain to systemic toxins and inflammatory mediators (102, 103). This sustained process is implicated in progressive synaptic loss, white matter damage, and cognitive decline, thereby contributing to the long-term burden of PSCI (91, 103, 104).

## Evidence on the prediction of long-term cognitive outcomes based on inflammatory-marker trajectories

### Associations between inflammatory-trajectory subtypes and cognitive function

With the accumulation of longitudinal clinical datasets, researchers have moved beyond simple static evaluations to characterize the immense heterogeneity of inflammatory responses and cognitive recovery patterns following acute ischemic stroke (AIS). Applying advanced latent class growth analysis (LCGA) and growth mixture modeling (GMM) has revealed that post-stroke cognitive function does not follow a uniform, linear pathway of decline or recovery, but is instead divided into distinct, clinically relevant trajectories (66). To provide a structured overview of the current clinical landscape, the landmark longitudinal cohorts, targeted inflammatory biomarkers, temporal sampling protocols, trajectory modeling methods, and key neurocognitive outcomes are comprehensively synthesized in Table 1.

As summarized in this narrative synthesis (Table 1), large-scale prospective cohort studies provide robust clinical evidence for these heterogeneous pathways. For instance, the Stroke and Cognition (STROKOG) collaboration study analyzed short-term trajectories of post-stroke cognitive function using a pooled cohort of 1,149 patients (66). As detailed in Table 1, Latent Class Growth Analysis (LCGA) in the STROKOG study identified three distinct 1-year cognitive trajectories: Low-performance (17%), Medium-performance (48%), and High-performance (35%). The study established that advanced age, lower educational attainment, diabetes (Relative Risk Ratio [RRR] = 3.78), large-artery stroke (RRR = 2.77), and severe/moderate stroke (RRR = 3.17) were major risk factors for belonging to the Low-performance trajectory.

Large-scale epidemiological studies have provided a robust clinical foundation for these observations. The Norwegian Cognitive Impairment After Stroke (Nor-COAST) study, through a series of prospective analyses, tracked systemic inflammatory biomarkers and longitudinal cognitive trajectories (21, 22). In their 2025 analysis of 466 ischemic stroke survivors (Table 1), Sandvig et al. used group-based trajectory modeling (GBTM) to identify three distinct MoCA trajectories over 36 months: “Low & declining” (11%), “Moderate & stable” (33%), and “High & increasing” (56%) (21). Higher acute-phase values of the terminal complement complex (TCC), IL-6, macrophage inflammatory protein-1α (MIP-1α), neopterin, quinolinic acid (QA), and the 4-pyridoxic acid ratio (PAr) were significantly associated with the “Low & declining” group, while acute-phase TNF and IL-8 predicted progressive 36-month MoCA decline, particularly in patients with pre-stroke cognitive impairment. This aligns with their 2023 study on 455 Nor-COAST participants (Table 1), which demonstrated that elevated concentrations of seven baseline biomarkers (TCC, IL-6, MIP-1α, IL-1Ra, TNF, IL-7, IL-8) were significantly associated with lower MoCA scores at 36 months, with TCC, IL-6, and MIP-1α exhibiting persistent negative associations across all follow-up waves (3, 18, and 36 months) (22).

Similarly, the ICONS study (*N* = 1,003), a prespecified prospective substudy of the Impairment of CognitiON and Sleep after acute ischemic stroke or TIA (Table 1), demonstrated that elevated baseline Interleukin-6 (IL-6) was an independent predictor of 1-year cognitive decline (defined as a MoCA reduction of ≥2 points between 3 months and 1 year, which occurred in 23.73% of the cohort) (54). Specifically, patients in the highest quartile of baseline IL-6 (≥4.29 ng/L) exhibited a 1.95-fold higher risk of cognitive decline than those in the first quartile (adjusted OR = 1.95, 95% CI: 1.13–3.38, *p* = 0.0167), proving that acute IL-6 levels predict 1-year cognitive outcome (54).

**Figure 4:**
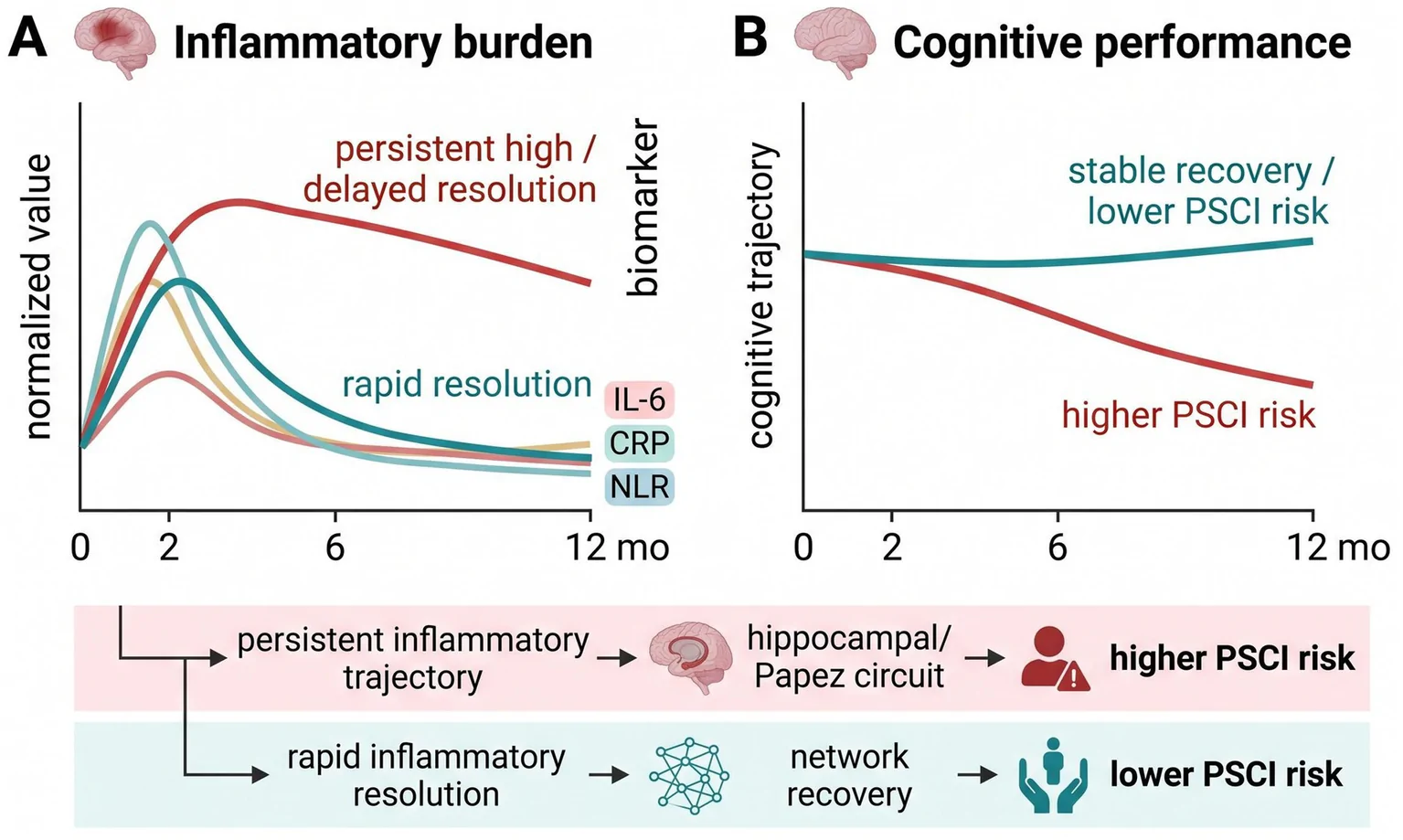
Inflammatory-trajectory patterns and long-term cognitive outcomes after stroke. **(A)** Depicts inflammatory-burden trajectories, contrasting a persistent-high/delayed-resolution pattern with a rapid-resolution pattern using IL-6, CRP, and NLR as representative biomarker domains. **(B)** Depicts corresponding cognitive trajectories, linking persistent inflammatory burden to higher PSCI risk and progressive decline, and rapid inflammatory resolution to stable recovery and lower PSCI risk. The lower summary strip connects residual inflammation with hippocampal/Papez-circuit vulnerability and supports trajectory-guided prevention and follow-up strategies. Source, software, and provenance: This author-directed conceptual illustration was based on the published clinical and mechanistic literature cited in the corresponding main-text section and mapped in Supplementary file S1. The curves are illustrative trajectory archetypes and are not fitted data from a single cohort or validated clinical thresholds. No patient-level data, original experimental images, or quantitative datasets are depicted. An initial visual draft was generated from an author-written text specification using OpenAI GPT-image, version 2.0 (OpenAI Media Service API). The authors independently verified and approved the final scientific content, labels, arrows, and graphical relationships. The complete prompt and materials inventory are provided in Supplementary file S1 and the accompanying plain-text prompt record.

To visually grasp this clinical phenomenon, we have mapped these prognostic paradigms in Figure 4, which contrasts inflammatory burden and cognitive performance across a 12-month timeline. As illustrated in Figure 4A, patients classified under the “Persistent High/Delayed Resolution” trajectory subtype (characterized by an immediate post-stroke surge of IL-6, CRP, and NLR that fails to return to baseline by month 3) show persistently elevated inflammatory burden, whereas the rapid-resolution subtype displays a steep post-acute decline in inflammatory burden. In Figure 4B, the persistent-high trajectory is paired with progressive cognitive decline and higher PSCI risk, while the rapid-resolution trajectory is paired with stable or improving cognitive performance and lower PSCI risk. The lower summary strip in Figure 4 further links residual inflammatory trajectories to hippocampal/Papez-circuit vulnerability and higher PSCI risk.

### Predictive value of dynamic inflammatory parameters for cognitive impairment

In clinical risk stratification, dynamic parameters derived from longitudinal monitoring—such as the rate of biomarker clearance, the area under the curve (AUC), the time-to-peak, and the presence of subacute elevations—provide superior prognostic value compared to traditional, static baseline measurements (105, 106). This is particularly evident in studies investigating the neutrophil-to-lymphocyte ratio (NLR). While admission NLR is a useful indicator of acute stress, the post-revascularization NLR trajectory (specifically the rate of decline within 48–72 h) is a strong independent predictor of 90-day clinical outcomes and long-term cognitive function (105, 107). A slow decline or secondary spike in the NLR trajectory indicates a persistent, unmitigated immune response, which is associated with blood–brain barrier (BBB) damage, symptomatic intracranial hemorrhage, and subsequent post-stroke cognitive impairment (PSCI) (108, 109). This clinical utility is also observed in other inflammatory contexts, where dynamic, non-linear fluctuations in peripheral inflammatory profiles are more closely associated with neurological deterioration and poor outcomes than static measurements (106).

The superior predictive value of these dynamic parameters has led to the adoption of advanced machine learning (ML) frameworks, which are summarized in the five-stage methodological pipeline shown in Figure 3. Traditional regression analyses often struggle to capture non-linear interactions and complex temporal dependencies in longitudinal datasets. To address these analytical limitations, Table 2 provides a multidimensional, critical comparison between traditional latent class statistical modeling (e.g., GCM, LCGA, GMM) and advanced non-parametric machine learning architectures (e.g., XGBoost, Random Forest) in terms of their mathematical assumptions, handling of high-dimensional longitudinal trajectories, and predictive accuracy for PSCI. As delineated in this comparative matrix (Table 2), while latent class models excel in capturing population-level heterogeneity and defining distinct clinical phenotypes, machine learning models like XGBoost demonstrate significantly higher predictive accuracy (AUROC) by processing complex, non-linear interactions.

This clinical predictive superiority is demonstrated by Yang et al. (110) in their 2026 study of 261 acute ischemic stroke patients within 7 days of onset (Table 1). Using LassoCV for feature selection, they identified serum vascular endothelial cadherin (VE-Cad), NIHSS score, age, drinking history, CRP, and education years as key predictors of 3-month PSCI (defined as MoCA < 26). When modeled via Extreme Gradient Boosting (XGBoost), VE-Cad emerged as the strongest predictor of PSCI risk. The XGBoost model demonstrated outstanding performance (validation set AUC = 0.806, sensitivity = 85.0%, accuracy = 73.4%), significantly outperforming traditional logistic regression (LR). SHAP force plots revealed that individual PSCI risk surged exponentially when serum VE-Cad exceeded 13.64 ng/mL and CRP exceeded 10.27 mg/L (Table 1). This clinical risk stratification is further supported by the Residual Inflammatory Risk (RIR) Cohort study conducted by Gang et al. (82) in 2025 on 172 AIS patients (Table 1). Six months post-stroke, 33.7% of the cohort developed PSCI (MoCA < 22). Patients with RIR (defined as LDL-C < 2.6 mmol/L and hs-CRP ≥ 2.0 mg/L) exhibited a 4.496-fold increased risk of PSCI (OR = 4.496, 95% CI: 1.157–17.477, *p* = 0.030) after adjusting for major confounders, while the combination of residual cholesterol and inflammatory risk (RCIR) further elevated the risk (OR = 7.357, 95% CI: 2.081–26.006, *p* = 0.002).

### Heterogeneity and limitations in existing studies

Despite the clinical promise of dynamic inflammatory trajectories, the translation of these findings into routine clinical practice is limited by significant heterogeneity in study designs and methodology. A systematic review of longitudinal stroke cohorts highlights substantial variation in the choice of inflammatory biomarkers, blood sampling intervals, and cognitive assessment tools. For instance, while some studies assess cognitive function using brief screening instruments such as the Mini-Mental State Examination (MMSE), which is still widely used in many clinical settings, others employ the more sensitive Montreal Cognitive Assessment (MoCA) or comprehensive neuropsychological batteries (111, 112). This lack of standardization introduces diagnostic bias, limits cross-cohort comparisons, and complicates meta-analytical validation (113). Furthermore, many current clinical investigations suffer from methodological limitations, including relatively small sample sizes and the lack of healthy, age-matched control groups, which restricts the generalizability of the identified trajectory profiles (22, 114).

Additionally, the biological specificity of circulating inflammatory markers remains a challenge. Peripheral blood concentrations of CRP or IL-6 are highly sensitive but relatively non-specific, as they are easily influenced by age, systemic comorbidities (such as subclinical chronic infections or autoimmune diseases), and procedural stress (115–117). A major limitation of current clinical studies is the insufficient simultaneous tracking of a broader panel of relevant pathways, such as glial activation markers or specific neuroinflammatory mediators. Without a more comprehensive, multi-marker approach, peripheral systemic assessments may fail to accurately reflect central neuroinflammatory processes (118). Standardizing longitudinal protocols—including high-frequency, standardized sampling schedules, comprehensive cognitive testing, and multi-marker panels—is therefore essential to advance the field and improve the clinical utility of trajectory-guided risk prediction (21, 22).

## Discussion

This review synthesizes current evidence regarding the dynamic temporal trajectories of inflammatory biomarkers following acute ischemic stroke and their relationship with long-term post-stroke cognitive impairment (PSCI). Pathophysiologically, the post-stroke immune response is highly dynamic, transitioning from an acute tissue-damage reaction to a subacute resolution phase, and in many patients, into a state of chronic, low-grade neuroinflammation (82). This continuous evolution is more accurately captured by longitudinal trajectory modeling than by static, single-time-point measurements, as evidenced by studies linking acute-phase inflammatory markers to PSCI (9, 107, 119). Pathophysiological cascades within the neurovascular unit, particularly the synergistic interaction between inflammatory mediators and vascular components, play a key role in driving BBB disruption. Elevated circulating inflammatory cytokines, such as interleukin-18 (IL-18) and matrix metalloproteinase-9 (MMP-9), are independently associated with PSCI and may mediate its development through sustained vascular and inflammatory pathways (98). This disruption facilitates the infiltration of peripheral immune cells and sustains a persistent neuroinflammatory state, characterized by chronic microglia activation (120). This prolonged inflammation contributes to hippocampal damage and synaptic dysfunction, ultimately leading to cognitive decline (121).

To establish the clinical and epidemiological relevance of these pathways, this review integrates findings from major prospective cohorts, including the Nor-COAST (22) and related longitudinal cohorts (21, 54) (summarized in Table 1). These cohorts demonstrate that longitudinal trajectories of markers such as IL-6 and CRP are more robust predictors of cognitive decline than static baseline levels, highlighting the importance of the duration and resolution of the inflammatory response. Furthermore, to move beyond association and investigate causality, researchers are increasingly utilizing Mendelian Randomization (MR) studies. By using genetic variants as instrumental variables, MR analyses have provided evidence that genetically determined systemic inflammatory pathways—specifically the IL-6 signaling cascade—are linked to cognitive outcomes (122, 123). This genetic validation, supported by findings from bidirectional MR analyses on chronic inflammation and intrinsic capacity (122), suggests that persistent post-stroke inflammation is not merely a marker of tissue damage, but a direct causal driver of progressive neurodegeneration and cognitive impairment (124, 125).

Translating these insights into clinical practice requires advanced computational tools to improve risk prediction. Comparing modern machine learning algorithms (such as XGBoost and Random Forest) against traditional statistical methods (such as LCGA and logistic regression) shows that ML models are better able to capture complex, non-linear interactions within longitudinal datasets, yielding superior predictive accuracy for PSCI (as detailed in Table 2) (110). Identifying patients with high-risk inflammatory trajectories or high Residual Inflammatory Risk (RIR) (82) provides an opportunity for targeted clinical interventions. Patients exhibiting persistently elevated inflammatory profiles or delayed resolution could be selected for personalized secondary prevention trials. These might include targeted anti-inflammatory therapies (such as colchicine, IL-1β inhibitors, or IL-6 receptor antagonists) combined with intensive cognitive rehabilitation, aiming to break the chronic neuroinflammatory loop and preserve long-term cognitive function.

## Conclusion

In conclusion, the post-stroke inflammatory response is a dynamic, multi-phase process that is closely linked to long-term cognitive outcomes. Longitudinal trajectory modeling, using statistical frameworks (LCGA/GMM) and machine learning algorithms (XGBoost), has demonstrated that persistent, low-grade systemic inflammation and delayed resolution are associated with post-stroke cognitive impairment (PSCI). Pathophysiologically, this decline is driven by an ongoing inflammatory cascade involving microglia activation, blood–brain barrier disruption (mediated by mCRP/VE-cadherin interaction), and secondary neurodegeneration within critical cognitive networks. Epidemiological data from cohorts such as Nor-COAST, STROKOG, and ICONS, alongside causal validation from Mendelian Randomization studies, support the role of persistent inflammation as a key driver of cognitive decline. Future research should focus on conducting large-scale, prospective studies with standardized longitudinal sampling, multi-marker panels, and advanced computational modeling. These efforts are needed to validate risk-prediction models and guide personalized, anti-inflammatory therapeutic trials to mitigate the burden of PSCI and improve long-term outcomes for stroke survivors.

## Supplementary material

The Supplementary material for this article can be found online at:

https://www.frontiersin.org/articles/10.3389/fneur.2026.1903189/full#supplementary-material

## Glossary

AIS: Acute ischemic stroke
ANXA1: Annexin A1
ApoE: Apolipoprotein E
ARE: Antioxidant response element
AUC: Area under the curve
AUROC: Area under the receiver operating characteristic curve
BBB: Blood–brain barrier
BDNF: Brain-derived neurotrophic factor
CNS: Central nervous system
CRP: C-reactive protein
DAMPs: Danger-associated molecular patterns
FPR2/ALX: Formyl peptide receptor 2/lipoxin A4 receptor
GBTM: Group-based trajectory modeling
GCM: Growth curve model
GMM: Growth mixture modeling
HCY: Homocysteine
HKr: 3-Hydroxykynurenine ratio
HMGB1: High-mobility group box 1
HPA axis: Hypothalamic–pituitary–adrenal axis
hs-CRP: High-sensitivity C-reactive protein
IL: Interleukin
IL-1β: Interleukin-1 beta
IL-6: Interleukin-6
IL-8: Interleukin-8
IL-17A: Interleukin-17A
IL-18: Interleukin-18
IRI: Ischemia–reperfusion injury
IVT: Intravenous thrombolysis
LCGA: Latent class growth analysis
LGM: Latent growth model
LPS: Lipopolysaccharide
LTP: Long-term potentiation
LVO: Large vessel occlusion
mCRP: Monomeric C-reactive protein
ML: Machine learning
MMP-9: Matrix metalloproteinase-9
MoCA: Montreal Cognitive Assessment
MR: Mendelian randomization
NETs: Neutrophil extracellular traps
NF-κB: Nuclear factor kappa B
NIHSS: National Institutes of Health Stroke Scale
NLR: Neutrophil-to-lymphocyte ratio
NLRP3: NOD-like receptor protein 3
NOX: NADPH oxidase
Nrf2: Nuclear factor erythroid 2-related factor 2
pCRP: Pentameric C-reactive protein
PAr: 4-Pyridoxic acid ratio
Pic: Picolinic acid
PSCI: Post-stroke cognitive impairment
QA: Quinolinic acid
RCIR: Residual cholesterol and inflammatory risk
RIR: Residual inflammatory risk
ROS: Reactive oxygen species
SHAP: SHapley Additive exPlanations
SIIS: Stroke-induced immunosuppression
SNS: Sympathetic nervous system
TCC: Terminal C5b-9 complement complex
Th17: T helper 17
TIA: Transient ischemic attack
TLR: Toll-like receptor
TNF-*α*: Tumor necrosis factor-alpha
VE-Cad: Vascular endothelial cadherin
VCAM-1: Vascular cell adhesion molecule-1
XGBoost: Extreme Gradient Boosting
