# Supplementary material for: Dynamic trajectories of inflammatory biomarkers and post-stroke cognitive impairment: a comprehensive review of neuroimmune mechanisms, longitudinal modeling, and clinical translation
Source: Front Neurol. 2026 Aug 20;17:1903189. doi: 10.3389/fneur.2026.1903189 (PMC13539562; doi:10.3389/fneur.2026.1903189)
Supplement: Supplementary file 1 [file Data_Sheet_2.DOCX]

**Supplementary File S1**

**Full provenance, source mapping, and prompt record for AI-assisted conceptual figures**

Manuscript ID: 1903189

Manuscript: Dynamic Trajectories of Inflammatory Biomarkers and Post-Stroke Cognitive Impairment: A Comprehensive Review of Neuroimmune Mechanisms, Longitudinal Modeling, and Clinical Translation

# **Purpose and Scope**

This supplement responds to the request for complete transparency regarding the use of generative AI in the preparation of Figures 1–4 and the graphical abstract. It records the image-generation software and version, the complete author-written prompts/instructions, all input-material categories, figure-by-figure literature-source mapping, the generation date, available technical provenance, final-output identifiers, and the authors’ verification procedure. The identical verbatim prompts are also supplied as a plain-text companion file, and the final submitted image identifiers are provided in a machine-readable CSV provenance manifest.

*All visual items are author-directed conceptual schematics. They do not contain patient-level data, original experimental images, unpublished datasets, or quantitative data plots. In particular, the curves in Figure 4 are illustrative trajectory archetypes and are not data from a single cohort.*

# **Software and Technical Record**

| **Image-generation tool** | OpenAI gpt-image, version 2.0. |
| --- | --- |
| **Service** | OpenAI Media Service API. |
| **Initial draft date** | 7 July 2026. The model and service labels were transcribed from the retained technical record supplied by the authors. |
| **Output export record** | Where retained, related PDF export metadata identify GPL Ghostscript 9.53.3. This is an export utility, not the image-generation system. |
| **Generation parameters** | The service did not expose a seed, temperature, sampler, or model-specific generation log; no such parameters were retained. |
| **Final file identifiers** | The accompanying CSV provenance manifest reports the submitted filename, visual item, pixel dimensions, file size, and SHA-256 hash for each final JPEG image. |

# **Complete Input-Material Inventory**

| **Text inputs** | Author-written specifications for each conceptual figure. The complete unabridged instructions are reproduced below and supplied as an accompanying plain-text file. |
| --- | --- |
| **Scientific source materials** | Published literature cited in the manuscript, mapped by figure below. The literature informed the scientific content; it was not uploaded as visual input. |
| **Reference images** | None. No third-party reference images or external visual assets were supplied as inputs. |
| **Clinical or experimental inputs** | None. No patient images, participant-level data, original experimental images, or unpublished datasets were supplied or used. |
| **Quantitative inputs** | None. No numerical data file was supplied to generate any visual. Figure 4 trajectory lines are illustrative rather than measurements from a single cohort. |

# **Figure-by-Figure Literature Source Map**

Citation numbers refer to the revised manuscript. The cited literature supports the conceptual scientific content, not the graphical style or image-generation process.

| **Figure** | **Conceptual content** | **Literature source map** | **Data status** |
| --- | --- | --- | --- |
| Figure 1 | Focal ischemic injury; DAMP/HMGB1/TLR4 signaling; microglial/NLRP3 activation; peripheral immune entry; ANXA1/FPR2-ALX resolution signaling; hippocampal/Papez-circuit injury. | Manuscript references 3, 9-11, 29, 31-36, and 41-47. | Conceptual schematic only; no patient-level, experimental, or quantitative dataset is plotted. |
| Figure 2 | mCRP-VE-cadherin axis and BBB disruption; outside-in endothelial injury and inside-out glial-parenchymal feedback. | Manuscript references 27, 41-47, 57, and 109. | Conceptual schematic only; no patient-level, experimental, or quantitative dataset is plotted. |
| Figure 3 | Five-stage workflow: sampling, harmonization, trajectory modeling, prediction models, and clinical translation. | Manuscript references 17, 66-72, and 105-110. | Conceptual workflow only; no cohort data or model output is plotted. |
| Figure 4 | Illustrative persistent-high/delayed-resolution and rapid-resolution inflammatory patterns with paired cognitive-outcome trajectories. | Manuscript references 9, 15-16, 21-24, 54, 82, and 105-110. | Illustrative curves, not data from a single cohort; no quantitative dataset is plotted. |
| Graphical abstract | Integrated conceptual summary of ischemic stroke, immune activation, BBB disruption, trajectory modeling, and PSCI risk stratification. | Conceptual synthesis of the literature categories mapped above and cited in the manuscript. | Conceptual illustration only; no patient-level, experimental, or quantitative dataset is plotted. |

# **Author Verification and Responsibility**

The authors independently checked the final scientific claims, labels, citations, and visual content against the cited literature. Generative AI was not used to generate study data, perform analyses, select references, or establish scientific conclusions. The authors take full responsibility for the manuscript and all accompanying visual materials.

**Full Author-Written Prompts / Instructions**

The following are the complete textual specifications supplied for the initial visual drafts. No image input accompanied these instructions. The identical prompts are supplied separately in the plain-text companion file Frontiers_Neurology_R2_Supplementary_File_S1_Full_Prompts_FINAL.txt.

## **Figure_1_ImageGen**

Use case: scientific-educational infographic diagram. Asset type: Figure 1 for a Frontiers in Neurology clinical review. Create a high-resolution landscape 16:9 scientific mechanism figure on a pure white background, high-detail vector BioRender style, polished top medical journal appearance. Title text: "Post-stroke central and systemic inflammatory cascade". Composition: five clearly separated labeled panels A-E connected by directional arrows. Panel A: "Focal ischemic injury" with a brain ischemic lesion icon and compact molecular labels "DAMPs", "HMGB1", "TLR4". Panel B: "Microglial activation" with activated microglia, NLRP3 inflammasome, labels "IL-1B" and "IL-18". Panel C: "Peripheral immune entry" with blood vessel / BBB interface, neutrophils, Th17 cells, label "IL-17A". Panel D: "Resolution signaling" with ANXA1, FPR2/ALX, resolving microglia/macrophage phenotype, shown as a counter-regulatory green pathway. Panel E: "Cognitive network injury" with hippocampus, Papez circuit network disruption, and label "PSCI". Visual logic: A -> B -> C -> E as the pro-inflammatory injury pathway; B -> D -> E as the resolution-modulating pathway. Use blue for brain injury, purple for microglia, orange/red for peripheral inflammation, green for resolution signaling, magenta for cognitive injury. Keep labels large, crisp, and readable. Avoid the labels "ischemic penumbra" and "reperfusion ROS". Clean unbranded export, no source labels or reference numbers.

## **Figure_2_ImageGen**

Use case: scientific-educational infographic diagram. Asset type: Figure 2 for a Frontiers in Neurology clinical review. Create a high-resolution landscape 16:9 neurovascular-unit mechanism figure on a pure white background, high-detail vector BioRender style, polished top medical journal appearance. Title text: "mCRP-VE-cadherin axis and BBB disruption after stroke". Overall layout: a horizontal neurovascular unit with blood lumen at the top, endothelial-cell layer with VE-cadherin adherens junctions in the middle, basement membrane below, and brain parenchyma at the bottom. Use a clear central divider showing two directional mechanisms. Left/top pathway, label: "Outside-in pathway". Show circulating pCRP in the blood lumen converting into mCRP on injured endothelial membranes; mCRP binds VE-cadherin; VE-cadherin junctions loosen; endothelial gaps increase BBB permeability. Required labels: "pCRP", "mCRP", "VE-cadherin binding", "junction loss", "increased permeability". Right/bottom pathway, label: "Inside-out pathway". Show brain parenchymal inflammation feeding back to the barrier: activated microglia, reactive astrocyte end-feet, pericyte injury, infiltrating leukocyte, MMP-9, cytokines and ROS damaging endothelial junctions from the abluminal side. Required labels: "astrocyte end-feet", "pericyte", "microglia", "MMP-9", "cytokines", "ROS", "brain parenchyma". Visual style: clean arrows with opposing directions, red/orange for outside-in vascular inflammatory injury, purple/green for inside-out glial-parenchymal feedback, blue for endothelium, lavender basement membrane. Make all text large and readable, no crowded labels. Clean unbranded export, no source labels or reference numbers.

## **Figure_3_ImageGen**

Use case: scientific-educational workflow diagram. Asset type: Figure 3 for a Frontiers in Neurology clinical review. Create a high-resolution landscape 16:9 methodology workflow figure on a pure white background, high-detail vector BioRender / top medical journal style. Title text: "Five-stage workflow for inflammatory-trajectory analysis and PSCI prediction". Main flow: five large numbered stages in a left-to-right timeline, each with a distinct color and medical/scientific icon. Stage 1: "Longitudinal sampling" with blood tubes, clock icons, and time labels "admission", "48-72 h", "14 d", "3 mo". Stage 2: "Data harmonization" with database/table icon and covariate labels "infection", "reperfusion", "stroke severity", "medications". Stage 3: "Trajectory modeling" with multi-curve plot icon and model labels "LCGA", "GMM", "GBTM". Stage 4: "Prediction models" with algorithm/network icon and labels "XGBoost", "Random Forest", "SHAP". Stage 5: "Clinical translation" with patient stratification icon and labels "PSCI risk groups", "trial enrichment", "personalized follow-up". Lower section: split from Stage 3 into two branches. Left branch: "Parametric branch" with label "interpretable latent classes". Right branch: "Machine-learning branch" with label "individualized risk". Both branches reconnect toward Stage 5. Visual requirements: exact stage numbers 1, 2, 3, 4, 5 must be visible; clear arrows; no irrelevant decorative elements; large readable sans-serif labels; blue/teal/purple/red/orange palette; balanced spacing. Clean unbranded export, no source labels or reference numbers.

## **Figure_4_ImageGen**

Use case: scientific-educational clinical infographic. Asset type: Figure 4 for a Frontiers in Neurology clinical review. Create a high-resolution landscape 16:9 publication-ready figure on a pure white background, high-detail BioRender / top medical journal style. Title text: "Inflammatory-trajectory patterns and long-term cognitive outcomes after stroke". Layout: two main panels side by side with clean axes and small biological icons. Panel A title: "A Inflammatory burden". Show normalized inflammatory-burden trajectories over time from 0 to 12 months. X-axis labels: "0", "2", "6", "12 mo". Y-axis label: "normalized value". Draw three or four colored biomarker trajectory curves but emphasize two archetypes: red curve "persistent high / delayed resolution" and teal curve "rapid resolution". Add small labels for "IL-6", "CRP", "NLR" near the biomarker legend. Panel B title: "B Cognitive performance". Show cognitive trajectory over the same time scale. Red curve declines over time and is labeled "higher PSCI risk". Teal curve remains stable or improves slightly and is labeled "stable recovery" / "lower PSCI risk". Add a small lower summary strip connecting the panels: persistent inflammatory trajectory -> hippocampal/Papez circuit vulnerability -> higher PSCI risk; rapid inflammatory resolution -> network recovery -> lower PSCI risk. Use brain and hippocampus icons, patient risk icons, and a concise legend. Visual style: large readable sans-serif labels, clean axes, high contrast red versus teal with colorblind-friendly support, no data points implying exact measured values, no clutter, balanced margins. Clean unbranded export, no source labels or reference numbers.

## **Graphical_Abstract_ImageGen**

Use case: scientific-educational graphical abstract. Asset type: graphical abstract for a Frontiers in Neurology clinical review. Create a high-resolution landscape 16:9 graphical abstract on a pure white background, high-detail vector BioRender style, polished top English medical journal appearance. Title text: "Dynamic inflammatory trajectories after stroke guide PSCI risk stratification". Composition: five-column left-to-right clinical flow with large icons, clean arrows, and concise labels. Column 1 title: "Acute ischemic stroke". Show occluded cerebral artery, ischemic brain lesion, and label "brain injury". Column 2 title: "Immune activation". Show activated microglia, neutrophil, lymphocyte, cytokine beads; labels "IL-6", "CRP", "NLR". Column 3 title: "BBB disruption". Show endothelial cells, VE-cadherin junction loss, mCRP, MMP-9, astrocyte end-feet and pericyte; labels "mCRP", "VE-cadherin loss", "MMP-9". Column 4 title: "Trajectory modeling". Show biomarker curves over time with labels "48-72 h", "14 days", "3 months", and four curve archetypes: "persistent high", "secondary spike", "moderate", "rapid resolution". Column 5 title: "PSCI risk". Show patient risk strata and brain/cognitive icons with labels "high risk", "moderate risk", "low risk"; add lower box "Personalized prevention" with icons for anti-inflammatory strategy, infection prevention, neuroprotection, and cognitive rehabilitation. Visual style: clean clinical graphical abstract, white background, high contrast blue/red/teal/orange palette, standard biological icons, readable sans-serif labels, subtle panel borders, no clutter. Clean unbranded export, no source labels or reference numbers.
